# Audit of Missed Seizure Rate and Management at the Northamptonshire Healthcare (NHS) Foundation Trust ECT Clinic

**DOI:** 10.1192/bjo.2025.10638

**Published:** 2025-06-20

**Authors:** Sanaa Moledina

**Affiliations:** Northamptonshire Healthcare NHS Foundation Trust, Northampton, United Kingdom

## Abstract

**Aims:** Missed seizures (MS) are non-therapeutic, making their frequency a key measure of an ECT clinic’s efficacy. In our initial audit at the NHFT ECT clinic (October 2021–March 2023), an MS rate of 12.6%, a restimulation rate of 67.5%, and poor adherence to the stimulus dosing protocol were identified. To address this, staff training, discussions, and dose chart displays were introduced. A follow-up audit was then conducted to assess the impact of these interventions.

**Methods:** The audit was conducted retrospectively over 13 months (June 2023–July 2024). Data were collected on total treatments, patient demographics, and stimulus doses. According to the NHFT ECT protocol, MS is defined as stimulation that fails to produce motor convulsions or EEG activity. The protocol recommends that during the seizure-threshold (ST) determination phase, dosing should be titrated as per the stimulus dosing protocol, while in the treatment phase (TP), restimulation should involve a 10% dose increase. The MS rate was calculated as the ratio of MS to total stimulations, and the restimulation rate as the ratio of total restimulations to MS.

**Results:** The clinic administered 210 bilateral ECT treatments to 22 patients – predominantly middle-aged (40–60 years), 59.1% male, and 77% White/British. The MS rate decreased to 11.6%, and the restimulation rate increased to 81.5%. Of the 27 MS, 19 occurred in the ST determination phase, with 17 restimulated, while 8 were in the TP, with 5 restimulated. Compared with the initial audit, the ST phase saw MS decrease from 81% to 70%, with correct dosing improving from 36% to 88%. After establishing the ST, full compliance with the dosing protocol was achieved – contrasting with the earlier audit, where 84% either received the same or a slightly higher dose. In TP, the current audit achieved full compliance with the recommended 10% dose increase for all restimulations, whereas previously, 2/3 doses were suboptimally increased. Additionally, documentation of the protocol showed marked improvement.

**Conclusion:** Appropriate management of MS is vital, as they are linked to treatment failure and increased post-ECT side effects. Our interventions have improved protocol adherence, yet further progress is needed. The Bridgend ECT clinic has maintained an MS rate of ≤5% over six years. To achieve similar outcomes, we recommend that the NHFT ECT clinic restimulate rather than proceed with ‘doubtful’ seizures, as they often lead to MS. Additionally, restimulating at the same dose as the previous MS in subsequent sessions should be avoided, as it is ineffective.

